# Ladies project: large database in endometrial cancers for a personalized treatment

**DOI:** 10.1007/s11547-024-01940-6

**Published:** 2024-12-17

**Authors:** Rosa Autorino, Raffaella Michela Rinaldi, Gabriella Macchia, Mariangela Boccardi, Inga Mihoci Roshanian, Rita Sebastiani, Bianca Santo, Donatella Russo, Martina Ferioli, Anna Benini, Elisabetta Perrucci, Arcangela Raguso, Sabrina Cossa, Paolo Matteucci, Claudia Talocco, Lisa Vicenzi, Fabio Trippa, Lorena Draghini, Antonietta Augurio, Fiorella Cristina Di Guglielmo, Paola Cocuzza, Francesca Pistis, Francesca De Felice, Sofia Meregalli, Elisa Maria Bonetto, Maria Tamburo, Vittorio Bini, Andrea Vavassori, Maria Antonietta Gambacorta, Cynthia Aristei

**Affiliations:** 1 Department of Radiation Oncology, Fondazione Policlinico Universitario A. Gemelli IRCCS, Rome, Italy; 2 Responsible Research Hospital, Unità Operativa Di Radioterapia Oncologica ‘Molise ART’, Campobasso, Italy; 3 Ospedale L’Aquila U.O.S.D Radioterapia E Cardioradiologia D.U.-PO, L’Aquila, Italy; 4 Ospedale Vito Fazzi, U.O. Radioterapia Oncologica, Lecce, Italy; 5 Department of Experimental, Diagnostic and Speciality Medicine-DIMES, Alma Mater Studiorum-Bologna University, Bologna, Italy; 6 Radiation Oncology, IRCCS Azienda Ospedaliero-Universitaria Di Bologna, DIMES, Alma Mater Studiorum-Bologna University, Bologna, Italy; 7 Radiation Oncology Section, Department of Medicine and Surgery, University of Perugia and Perugia General Hospital, Perugia, Italy; 8 Fondazione Casa Sollievo Della Sofferenza IRCCS, S. Giovanni Rotondo, Italy; 9 Operative Research Unit of Radiation Oncology, Fondazione Policlinico Universitario Campus Bio-Medico, Rome, Italy; 10 S.C. Radioterapia Oncologica Az. Ospedaliera S. Maria, Terni, Italy; 11 Radioterapia Oncologica Ospedale Santissima Annunziata, Chieti, Italy; 12 Radiation Oncology Unit, S. Luca Hospital, Healthcare Company Tuscany Nord Ovest, Lucca, Italy; 13 S.C. Radioterapia PO “A. Businco” ARNAS Brotzu, Cagliari, Italy; 14 Department of Radiotherapy, Policlinico Umberto I “Sapienza” University of Rome, Rome, Italy; 15 Department of Radiotherapy, San Gerardo Hospital, Monza, Italy; 16 Department of Radiotherapy, Azienda Ospedaliera, Cannizzaro, Catania, Italy; 17 Radioterapia Istituto Europeo Di Oncologia, Milan, Italy

**Keywords:** Endometrial cancer, Radiotherapy, Radiation therapy, Interventional radiotherapy; Brachytherapy, Adjuvant treatments

## Abstract

**Purpose:**

To compare Italian use with current international guidelines and to evaluate oncological outcomes and toxicity patterns of adjuvant radiation therapy (RT) for endometrial cancer (EC) in Italian women.

**Materials and methods:**

To conduct a retrospective multicentre Italian study a large database was set up. Inclusion criteria were: accrual between 2010 and 2020, treatment with surgery, post-operative external beam RT (EBRT) and/or interventional radiotherapy (IRT) associated or not with adjuvant chemotherapy. Oncological outcomes, acute and late toxicities were analysed according to RT schedule and risk group.

**Results:**

A total of 1848 patients, from 16 Italian RT centres were enrolled (median age 65 years, range 27–88). All patients received post-operative RT associated with chemotherapy in 31%. Patients were stratified on the basis of standard risk factors (Bosse et al. in Eur J Cancer 51:1742–50, 2015). After merging intermediate and high-intermediate risk classes into one intermediate group and including advanced and oligometastatic disease in the high-risk group, the low-risk group encompassed 124 patients, the intermediate-risk 1140, and the high risk 576. No low-risk patient developed local relapse (LR). Multivariate analysis showed that intermediate risk patients had a 2.5-fold increased risk of LR if treated with IRT alone *vs* EBRT-IRT boost. RT schedule did not impact significantly on LR in high risk patients. All acute toxicity parameters were highest in patients who received EBRT with simultaneous integrated boost (EBRT-SIB) and lowest in patients who received only IRT (*p* < 0.0001). Late toxicity was highest patients who received EBRT-SIB and lowest in those who were given EBRT with sequential boost (*p *< 0.0001).

**Conclusions:**

This retrospective study showed that Italian administration of adjuvant RT for EC is in accordance with current international guidelines. IRT alone for low-risk patients and EBRT associated with vaginal IRT remain standard adjuvant approaches for EC.

## Introduction

Endometrial cancer, the most common gynecological malignancy, accounts for about 8–10% of all female neoplasms. Worldwide, it is the 4th most frequent cancer and cause of cancer death in women [1]. In the last decade its incidence has increased greatly in developed countries, rising from global estimates of 43,470 new cases and 7950 deaths in 2010 through 121,578 new cases and 29,638 deaths in 2018 to about 417,367 new cases and 97,370 deaths in 2020 [1]. Causes include older average age of patients and widespread obesity.

Standard treatment for endometrial cancer is hysterectomy with bilateral salpingo-oophorectomy, with or without sentinel lymph node biopsy and/or lymph node dissection and pelvic washing followed, if necessary, by adjuvant chemotherapy and/or radiation therapy. Factors indicating adjuvant therapy are: patient’s age, disease stage, tumour diameter, histology and grade, myometrial, cervical, stromal and lympho-vascular invasion (LVSI) and lymph node status [2]. Different combinations of these prognostic factors led to the establishment of risk categories requiring specific treatment options.

In 2020 the ESGO/ESTRO/ESP organizations [3] added the molecular classification of endometrial cancer to the prognostic risk stratification which, until then, had guided adjuvant therapy use and type (Table 1). The molecular classification includes the following profiles: p53-abnormal (p53abn), mismatch repair–deficient (MMRd), DNA polymerase epsilon-mutation (*POLE*-mut) and no specific molecular profile (NSMP). It has prognostic value, may guide adjuvant chemotherapy decisions and was reported to predict response to radiation therapy in stage I endometrioid endometrial cancer. In fact, omitting radiation therapy in POLE-mut endometrial cancer seemed safe, as no loco-regional recurrences were observed. In MMRd endometrial cancer loco-regional recurrence-free survival was similar after external beam radiotherapy (EBRT), interventional radiotherapy (IRT) and no adjuvant therapy, suggesting the benefits of RT were limited. In p53abn EBRT yielded a significantly better loco-regional recurrence-free survival than IRT. Finally, in NSMP, IRT was as effective as EBRT and significantly better than no adjuvant therapy for loco-regional tumor control [4].

**Table 1:** Patient stratification into prognostic risk groups, (modified 2020 ESGO/ESTRO/ESP prognostic risk groups) and current adjuvant treatments options

Risk groups used in this study	Risk groups	Cancer staging and features	Current adjuvant treatment options
Low	Low	Stage IA endometrioid, grade 1–2, LVSI negative or focal	No adjuvant treatment
Intermediate	Intermediate	Stage IB endometrioid, grade 1–2, LVSI negative or focal Stage IA endometrioid, grade 3, LVSI negative or focal Stage IA non-endometrioid (serous, clear cell, undifferentiated carcinoma, carcinosarcoma, mixed) without myometrial invasion	Adjuvant brachytherapy
	High-intermediate	Stage I endometrioid, substantial LSVI, regardless of grade and depth of invasion Stage IB endometrioid, grade 3, regardless of LVSI status Stage II	Adjuvant brachytherapy Adjuvant EBRT for substantial LVSI and/or for stage II Additional adjuvant chemotherapy for high-grade and/or substantial LVSI
High	High	Stage III–IVA with no residual disease Stage I–IVA non-endometrioid (serous, clear cell, undifferentiated carcinoma, carcinosarcoma, mixed) with myometrial invasion, and with no residual disease	Concurrent and adjuvant chemotherapy to radiotherapy or alternatively sequential chemotherapy followed by radiotherapy
	Advanced	Stage III–IVA with residual disease	Systemic therapy after surgical tumor deburking
	Metastatic	Stage IVB	Palliative treatment Tumor directed radiotherapy (e.g. SRT)

ESGO, European Society of Gynaecological Oncology; ESTRO European SocieTy for Radiotherapy and Oncology; European Society of Pathology (ESP)

Consequently, when opting for adjuvant therapy, adding molecular findings to prognostic risk factor stratification may improve disease control only in some subgroups of patients but others may be exposed to the risk of treatment-related side effects and worse quality of life. Furthermore, administering unnecessary treatments increases health care costs.

The present report describes a multi-centre Italian observational, retrospective study on adjuvant therapy in endometrial cancer. The LADIES (LArge Database In Endometrial cancerS) project set-up a large database to compare Italian use with current international guidelines [3] and evaluate outcomes and toxicity patterns.

## Materials and methods

This joint project was developed by two AIRO (Italian Association of Radiation and Clinical Oncology) study groups: the “Gynecology” and the “Brachytherapy, Interventional Radiotherapy, IORT”.

In step 1 a 5-question survey was sent to centres affiliated to the 2 study groups, inquiring about the number of patients with endometrial cancer that could be enrolled in a future database, treatments, involvement of a multi-disciplinary team in clinical case discussions and guidelines that were followed. Sixteen centres responded (Supplemental material), hypothesizing enrollment of about 800 patients.

In step 2, Group members (RA, AV, CA) drafted the protocol and database template, which were approved by the promoting centre (Fondazione Policlinico Universitario “A. Gemelli”, IRCCS, UOC Radioterapia Oncologica) and the Ethics Committees of participating centres.

Patients who were treated between 2010 and 2020 were included in the database. A minimum follow-up of 24 months was required.

All patients had undergone surgery, and, in accordance with risk stratification, were treated with EBRT and/or IRT**,** preceded or not by chemotherapy. Radiation treatment was one of the following: EBRT; EBRT with a sequential boost (EBRT-seq BOOST); EBRT with simultaneous integrated boost (EBRT-SIB); EBRT with boost delivered by IRT (EBRT–IRT BOOST); IRT.

Adjuvant external beam radiotherapy was delivered to the pelvis with volumetric modulated arc therapy strategy for a total dose of 45  Gy, 1.8  Gy/fraction. A concomitant (for a total dose of 55 Gy, 2.2 Gy/fraction) or sequential boost (additional 15–25 Gy) or brachytherapy boost (on vaginal cuff for a total dose of 10-15 Gy in 2/3 fractions weekly) were applied. High Dose Rate brachytherapy alone was delivered using Ir192 source, a single-tube cylindrical applicator placed into the vagina to cover with the prescribed isodose the upper 1/3 part of the vaginal mucosa at a depth of 5  mm, and an X-ray examination to plan the treatment. Small variations between the various centers were found.

### Variable definitions

In designing the database the following items were selected:

- Clinical parameters (age, BMI, comorbidity);
- Disease stage according to the 2009 and 2018 FIGO staging system. All patients that had been staged according to 2009 system were re-classified following the 2018 classification;
- Uterine surgery: A) Simple/Extra fascial Hysterectomy; B) Modified Radical Hysterectomy; C) Radical Hysterectomy; Other;
- Nodal surgery: unilateral pelvic lymph-node dissection; bilateral pelvic lymph-node dissection; para-aortic lymph-node dissection; lymph-node sampling; sentinel lymph-node biopsy
- Pathology: stage, grade, lymph node status, lymph vascular space invasion, stromal infiltration;
- Molecular biology;
- EBRT: total dose, dose per fraction, technique;
- IRT: total dose, dose per fraction, technique
- Chemotherapy: type and number of cycles;
- Acute toxicity with each treatment;
- Late toxicity;
- Oncological outcome: local relapse, distant metastasis-free survival, overall survival, cancer-specific survival.

Since molecular data were not available for most patients, the patients were classified on the basis of prognostic risk factors [3] merging the intermediate and high-intermediate risk classes into one intermediate group and designating the advanced and metastatic stages together as high-risk. The metastatic group included only 4 patients with oligometastatic disease at diagnosis who had received surgery (uterus, lymph nodes and metastatic site/s), chemotherapy and radiotherapy.

Table 1 shows patient stratification into low, intermediate and high risk groups.

### End points

The primary end-point was local relapse-free survival (LRFS) defined as the time from surgery to pelvic and/or vaginal relapse.

Secondary end-points were:

- Distant metastasis-free survival (DMFS), defined as the time from surgery to onset of distance recurrence.
- Overall survival (OS), defined as the time from surgery to latest follow-up or death, independently of the cause of death.
- Cancer-specific survival (CSS), defined as the time from surgery to death due to EC.
- Acute toxicity: gastrointestinal (GI), genitourinary (GU), hematological and cutaneous toxicity occurring during treatment and/or within 3 months of its ending. It was graded on the CTCAE v 5.0 scale.
- Late toxicity: GI/GU toxicity focusing on vaginal stenosis occurring 3 months or more after the end of treatment. Toxicity was graded on the CTCAE v 5.0 scale

Oncological outcomes (LRFS, DMFS, OS, CSS) were evaluated in each risk category according to radiation treatments.

### Statistical analysis

The Shapiro–Wilk test assessed whether variable distribution was normal. The Chi-square test with Yates’ continuity correction and Fisher’s exact test compared categorical variables. The Mann–Whitney's *U* test compared ordinal and non-normally distributed continuous variables. Survival curves were calculated by the Kaplan–Meier product-limit method, followed by a log-rank test to evaluate inter-group differences in event probabilities.

To examine risk factors affecting prognosis, univariate and multivariate Cox proportional-hazard regression models were fitted, incorporating all significant variables in the univariate analysis into the multivariate model.

Statistical analysis was performed using IBM-SPSS® version 26.0 (IBM Corp., Armonk, NY, USA, 2019). In all analyses, a two-sided *p*-value < 0.05 was considered significant.

## Results

A total of 1848 patients (median age 65 years, range 27–88 years) were enrolled. In 480 patients (25.9%) with available BMI, median BMI was 28.20 (range: 14.06–55.11). Multidisciplinary team data were available in 776 cases (41%) and the therapeutic approach was discussed in 419 (54%).

Complete datasets were available for age, tumor histology, grade and radiation treatment, and over 75% were complete for toxicity and outcomes.

Table 2 reports details of histology, tumour features, stages and missing data.

**Table 2:** Tumour features and classification (in bold the Figo 2018 tumour stages)

	Number of patients						Missing data
Histology							
Endometrial endometrioid Adenocarcinoma	1572						
Endometrial non endometrioid Adenocarcinoma	276						
Grading							58
G1	192						
G2	973						
G3	625						
LVSI							358
Negative	875						
Positive	615						
Emboli in cases of positive LVSI							222
Focal	158						
Diffuse	235						
FIGO 2018	**1**	**1a**	**1b**				
	8	263	771				
	**2**	**2a**	**2b**				
	357	18	35				
	**3**	**3a**	**3b**	**3c**	**3c1**	**3c2**	
	4	87	46	17	194	25	
	**4**	**4a**	**4b**				
	3	2	7				11

After surgery, all patients received adjuvant radiotherapy and 588 (31%) received adjuvant chemotherapy before radiotherapy. Table 3 reports radiotherapy schedules according to risk category.

**Table 3:** Distribution of patients according to Radiotherapy treatments, adjuvant chemotherapy (CT) and Risk stratification (number and percentage for each risk category)

Risk level	EBRT	EBRT-seq BOOST	EBRT-SIB	EBRT–IRT BOOST	IRT	Total	CT
Low risk	3 (2.4%)	1 (0.8%)	21 (16.9%)	16 (12.9%)	83 (66.9%)	124	7
Intermediate risk	115 (10.1%)	26 (2.3%)	136 (11.9%)	486 (42.6%)	377 (33.1%)	1140	161
High risk	95 (16.5%)	18 (3.1%)	147 (25.5%)	267 (46.4%)	49 (8.5%)	576	420
TOT	217 (11.7%)	45 (2.4%)	304 (16.4%)	772 (41.7%)	510 (27.5%)	1848	588 (31.8%)

EBRT, External beam radiotherapy; EBRT seq- BOOST, External beam radiotherapy with sequential boost; EBRT–SIB, External beam radiotherapy with simultaneous integrated boost; EBRT -IRT BOOST, External beam radiotherapy with interventional radiotherapy boost; IRT, Interventional radiotherapy

Notably, IRT alone was prevalent in low risk patients (67%). EBRT followed by IRT were administered to 43% of intermediate risk patients and to 46% of high risk cases.

### Local relapse

Local relapses occurred in 106/1761 patients (6%) with available information.

**Fig. 1:**
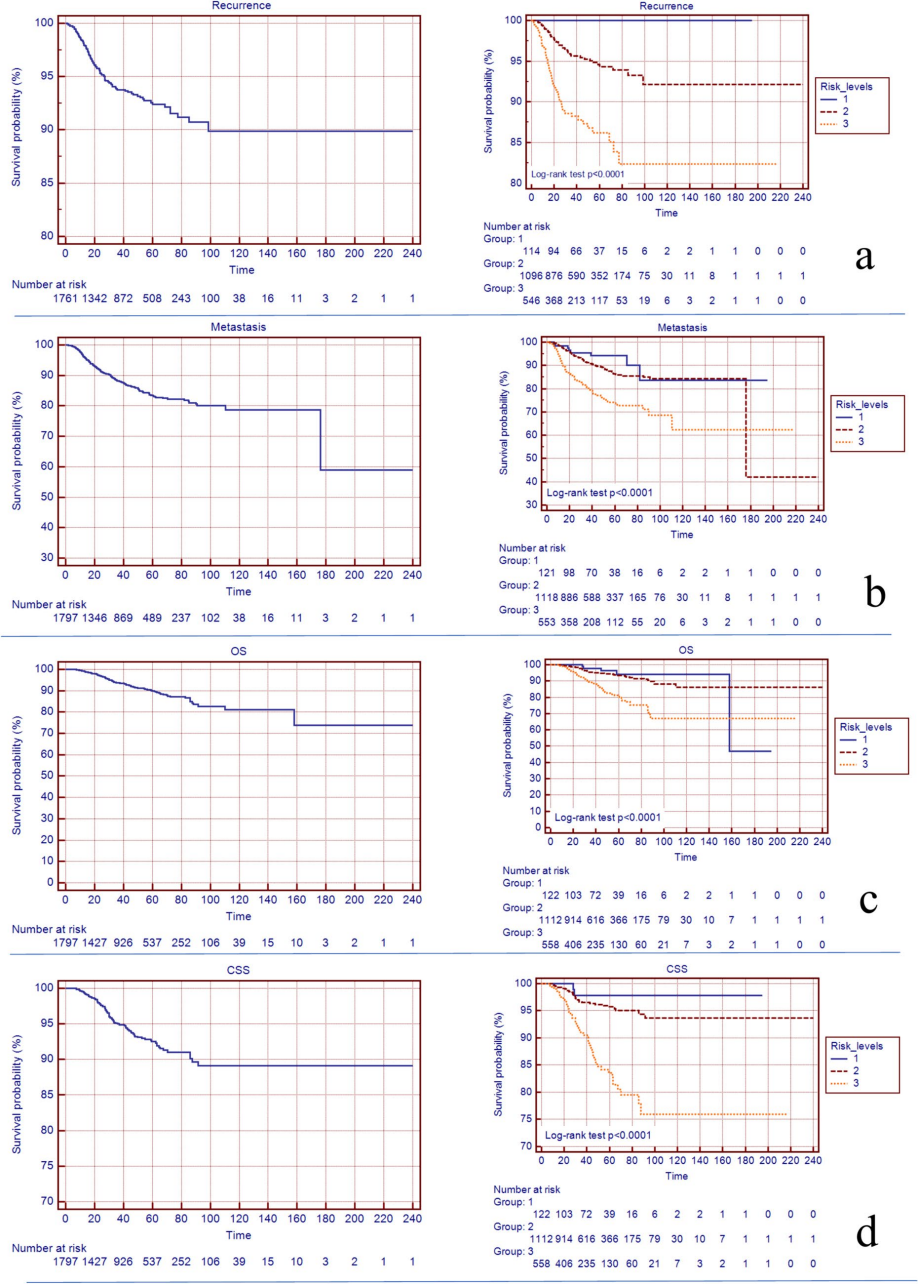
Kaplan–Meier curves for the local relapse-free survival (**a**), distant metastasis free survival (**b**), overall survival (OS) (**c**) and cancer specific survival (CSS) (**d**) for all patients (left) and for the three risk group levels (right)

Overall, the 5-year probability of local relapse-free survival was 92.4% (95%CI: 90.8–94). It was 100% (95%CI: 100–100) in the low-risk category, 94.3% (95%CI: 92.5–96.1) in the intermediate risk category and 86.2% (95%CI: 82.5–89.9) in the high risk category (*p* < 0.0001) (Fig. 1a).

Univariate analysis showed risk factors for local relapse were age (HR: 1.042; *p* < 0.0001), disease stage (HR: 1.150; *p* < 0.0001), grade (HR: 1.781; *p* = 0.001) and LVSI (HR: 1.768; *p* = 0.013) (Table 4).

**Table 4:** Risk factors correlation with outcomes through univariate and multivariate analysis

LR	Age	Univariate analysis	(HR:1.042; 95%CI: 1.020–1.065; *p* < 0.0001)
		Multivariate analysis	(HR:1.055; 95%CI: 1.029–1.082; *p* < 0.0001)
	Grade	Univariate analysis	(HR:1.781; 95%CI: 1.282–2.474; *p* = 0.001)
		Multivariate analysis	NS
	LVSI	Univariate analysis	(HR:1.768; 95%CI: 1.129–2.766; *p* = 0.013)
		Multivariate analysis	NS
	Disease Stage	Univariate analysis	(HR:1.150; 95%CI: 1.103–1.198; *p* < 0.0001)
		Multivariate analysis	(HR:1.134; 95%CI: 1.075–1.196; *p* < 0.0001)
Metastasys free survival	Age	Univariate analysis	(HR:1.030; 95%CI: 1.016–1.045; *p* < 0.0001)
		Multivariate analysis	(HR:1.042; 95%CI: 1.024–1.061; *p* < 0.0001)
	Grade	Univariate analysis	(HR:2.084; 95%CI: 1.650–2.631; *p* < 0.0001)
		Multivariate analysis	(HR:1.835; 95%CI: 1.391–2.422; *p* < 0.0001)
	LVSI	Univariate analysis	(HR:1.899; 95%CI: 1.397–2.581; *p* < 0.0001)
		Multivariate analysis	(HR:1.645; 95%CI: 1.181–1.2.291; *p* = 0.003)
	Disease Stage	Univariate analysis	(HR:1.092; 95%CI: 1.059–1.126; *p* < 0.0001)
		Multivariate analysis	(HR:1.077; 95%CI: 1.035–1.120; *p* < 0.0001)
OS	Age	Univariate analysis	(HR:1.083; 95%CI: 1.061–1.104; *p* < 0.0001)
		Multivariate analysis	(HR:1.103; 95%CI: 1.075–1.132; *p* < 0.0001)
	Grade	Univariate analysis	(HR:2.093; 95%CI: 1.563–2.801; *p* < 0.0001)
		Multivariate analysis	(HR:1.673; 95%CI: 1.162–2.409; *p* = 0.006)
	LVSI	Univariate analysis	(HR:1.517; 95%CI: 1.018–2.260; *p* = 0.040)
		Multivariate analysis	NS
	Disease Stage	Univariate analysis	(HR:1.115; 95%CI: 1.074–1.157; *p* < 0.0001)
		Multivariate analysis	(HR:1.131; 95%CI: 1.076–1.189; *p* < 0.0001)
CSS	Age	Univariate analysis	(HR:1.072; 95%CI: 1.047–1.098; *p* < 0.0001)
		Multivariate analysis	(HR:1.097; 95%CI: 1.065–1.131; *p* < 0.0001)
	Grade	Univariate analysis	(HR:2.615; 95%CI: 1.809–3.781; *p* < 0.0001)
		Multivariate analysis	(HR:2.090; 95%CI: 1.331–3.283; *p* = 0.001)
	LVSI	Univariate analysis	(HR:1.802; 95%CI: 1.126–2.884; *p* = 0.014)
		Multivariate analysis	NS
	Disease Stage	Univariate analysis	(HR:1.158; 95%CI: 1.109–1.208; *p* < 0.0001)
		Multivariate analysis	(HR:1.149; 95%CI: 1.086–1.216; *p* < 0.0001)

LR, local relapse; MFS, metastasis free survival; OS, overall survival; CSS, cancer specific survival

In multivariate analysis only age (HR: 1.055; *p* < 0.0001), and disease stage (HR: 1.134; *p* < 0.0001) were significant (Table 4).

Subdividing by risk classes and adding radiotherapy treatments to multivariate models, an increased risk of local recurrence was detected in intermediate risk patients who received IRT alone (HR: 2.513; 95%CI 1.222–5.169; *p* = 0.012) compared with the reference treatment (EBRT + IRT), while no differences were detected in high risk patients. The low risk class did not show any event and it was not possible to carry out the analysis.

### Distant metastasis

Distant metastasis occurred in 227/1797 patients (12.6%) with available information.

Overall, the 5-year probability of metastases-free survival was 83.4% (95%CI: 81.2–85.6). It was 94.1% (95%CI: 89.4–98.8), in the low-risk category, 86.4% (95%CI: 83.9–88.9) in the intermediate risk category and 74.1% (95%CI: 69.4–78.8) in the high risk category (*p* < 0.0001) (Fig. 1b).

In univariate analysis, risk factors for metastasis were age (HR: 1.030; *p* < 0.0001), disease stage (HR: 1.092; *p* < 0.0001), grade (HR: 2.048; *p* < 0.0001) and LVSI (HR: 1.899; *p* < 0.0001) (Table 4).

In multivariate analysis all variables remained significant (Table 4).

Analyzing the risk classes, in intermediate and high risk groups, EBRT-seq boost was associated with greater risk of metastases than reference treatment (EBRT- IRT) (HR: 2.719; 95%CI 1.143–6.467; *p* = 0.024 for intermediate risk group and HR: 2.696; 95% CI 1.097–6.624; *P* = 0.031 for high risk group). In the low risk class only 4 events happened and this made the multivariate model unstable, not allowing the analysis.

### Overall survival

Death from any cause was observed in 144/1797 patients (8%) with available information.

Overall, the 5-year probability of OS was 90% (95%CI: 88.2–91.8). It was 94% (95%CI: 87.9–100) in the low-risk category, 93.6% (95%CI: 91.8–95.4) in the intermediate-risk category and 80.7% (95%CI: 76.2–85.2) in the high-risk category (*p* < 0.0001) (Fig. 1c).

In univariate analysis, risk factors for OS were age (HR: 1.083; *p* < 0.0001), disease stage (HR: 1.115; *p* < 0.0001), grade (HR: 2.093; *p* < 0.0001) and LVSI (HR: 1.517; *p* = 0.040) (Table 4).

Significance persisted in multivariate analysis for age (HR: 1.103; *p* < 0.0001), disease stage (HR: 1.131; *p* < 0.0001) and grade (HR: 1.673; *p* = 0.006 (Table 4).

When OS in each risk category was correlated with RT type, no significant link emerged.

### Cancer specific survival

Cancer-specific deathoccurred in 100/1797 patients (5.6%) with available information.

Overall, the 5-year probability of CSS was 92.5% (95%CI: 90.9–94.1). It was 97.1% (95%CI: 94.9–100) in the low-risk category, 95.7% (95%CI: 94.3–97.1) in the intermediate-risk category and 83.6% (95%CI: 79.2–87.9). In the high-risk category (*p* < 0.0001) (Fig. 1d).

In univariate analysis, risk factors for CSS were age (HR: 1.072; *p* < 0.0001), disease stage (HR: 1.158; *p* < 0.0001), grade (HR: 2.615; *p* < 0.0001) and LVSI (HR: 1.802; *p* = 0.014) (Table 4).

In multivariate analysis, age (HR: 1.097; *p* < 0.0001), disease stage (HR: 1.149; *p* < 0.0001) and grade (HR: 2.090; *p* = 0.001) remained significant (Table 4).

When CSS in each risk category was correlated with RT type, no significance emerged.

### Toxicity

**Fig. 2:**
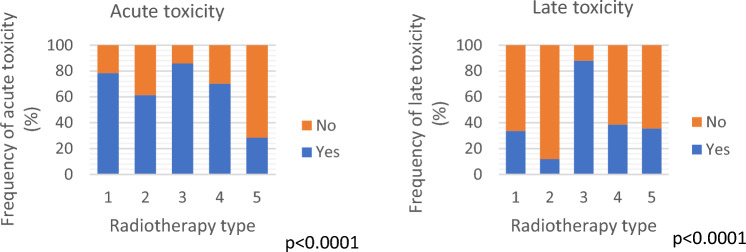
Acute (left panel) and late (right panel) toxicity incidence according to radiotherapy treatments. 1: EBRT = External Beam Radiotherapy treatment; 2: BOOST EBRT seq = External Beam Radiotherapy treatment with sequential boost; 3: EBRT + SIB = External Beam Radiotherapy treatment with concomitant boost; 4: EBRT + IRT = External Beam Radiotherapy treatment with Interventional radiotherapy boost; 5: IRT escl. = Interventional radiotherapy

Data on the incidence of acute and late toxicity related to the type of radiation treatment are shown in Fig. 2.

**Fig. 3:**
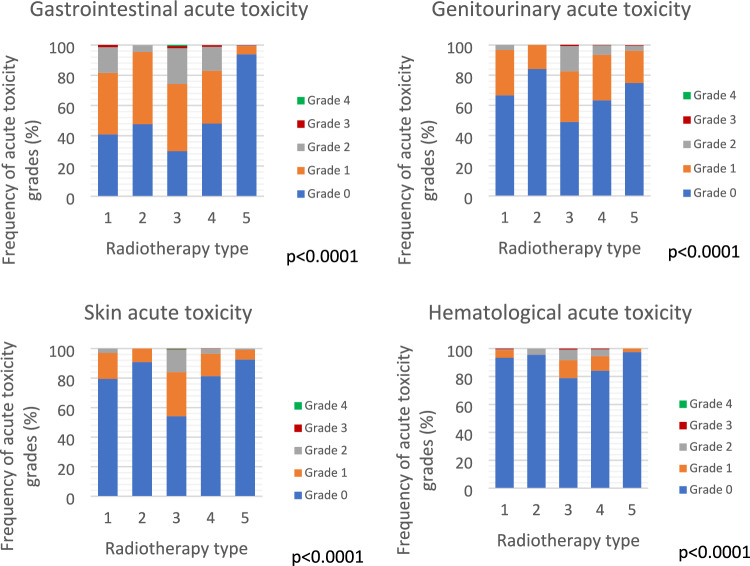
Acute toxicity according to radiotherapy treatments. 1: EBRT = External Beam Radiotherapy treatment; 2: BOOST EBRT seq = External Beam Radiotherapy treatment with sequential boost; 3: EBRT + SIB = External Beam Radiotherapy treatment with concomitant boost; 4: EBRT + IRT = External Beam Radiotherapy treatment with Interventional radiotherapy boost; 5: IRT escl. = Interventional radiotherapy

All acute toxicity parameters (genitourinary, gastrointestinal, skin and haematological) were highest in patients who received EBRT-SIB and lowest in patients who received only IRT (*p* < 0.0001; Fig. 3).

**Fig. 4:**
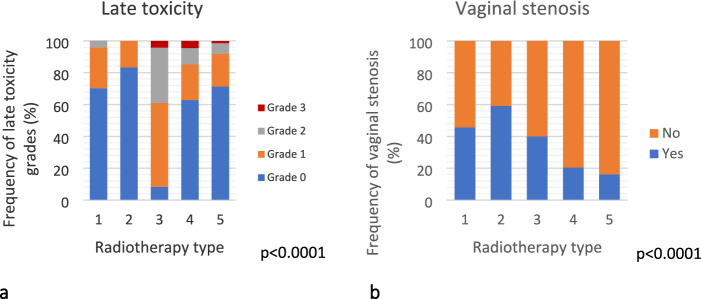
Late toxicity (panel **a**) and vaginal stenosis (panel **b**) incidence according to radiotherapy treatments. 1: EBRT = External Beam Radiotherapy treatment; 2: BOOST EBRT seq = External Beam Radiotherapy treatment with sequential boost; 3: EBRT + SIB = External Beam Radiotherapy treatment with concomitant boost; 4: EBRT + IRT = External Beam Radiotherapy treatment with Interventional radiotherapy boost; 5: IRT escl. = Interventional radiotherapy

Figure 4a shows late toxicity was highest patients who received EBRT-SIB and lowest in those who were given EBRT-seq Boost (*p* < 0.0001).

Figure 4b illustrates vaginal stenosis according to RT schedules showing highest incidence in patients who received EBRT-seq Boost.

## Discussion

To our knowledge, the present study is the first multicentre Italian initiative to recruit so many patients with endometrial cancer. As it accounts for 5–6% of female cancers nationwide in Italy, with 4,000 new cases per year [1] the objectives of the present study were to compare Italian use of adjuvant therapies with current guidelines [3], and to assess the impact of modern adjuvant radiotherapy on survival outcomes and toxicity profiles. To achieve an overview of treatments in the decade 2010–2020, a large database was set up and the results of the data analysis confirmed Italian Radiation Oncologists adhered to international guidelines.

The present study adopted a different risk stratification to what the guidelines report, merging the intermediate and high-intermediate risk classes into one intermediate group. The metastatic patients were pooled with advanced stage patients to constitute the high-risk category as the 4 oligometastatic patients had received surgery (uterus, lymph nodes and metastatic site/s) followed by chemotherapy and radiotherapy.

Initially, the ESMO risk classification [5, 6] did not include the adverse prognostic role of LVSI and tumour grade 3 within the intermediate-risk group (stage IA grade 3 or stage IB grade 1–2) which has since been recognized. Consequently, current guidelines for EC state that adjuvant therapy should be administered according to risk stratification [3]. No adjuvant therapy or only IRT is recommended as the gold standard of treatment for low-risk EC as EBRT did not increase local control or reduce the incidence of distant metastasis, and was associated with more toxicity, suggesting overtreatment. In the present low risk group, with 66.9% receiving only IRT, no case of local relapse occurred but 4 patients developed distant metastasis. Despite this, low risk EC was associated with good overall survival (94%) and cancer specific survival (97%), confirming the validity of IRT as adjuvant radiation therapy for these patients.

On the other hand, intermediate, high-intermediate, and high-risk groups were reported to require pelvic EBRT with a boost to the vaginal vault which, depending on risk factors, may be combined with chemotherapy [3]. EBRT alone was not effective as it was associated with a high relapse risk and low survival endpoints [7]. Overall, outcomes in the present cohort of intermediate risk patients, showed good probability of controlling local relapse and distant-metastasis (94% and 86%, respectively). The risk of local relapse did not, however, differ significantly after EBRT alone or EBRT + boost, probably because only 115 patients (< 10%) did not receive the boost. Furthermore, present results suggested boost timing (sequential, SIB or IRT) did not impact significantly on the risk of local relapse and distant metastasis. In our high risk patients the probabilities of relapse and metastasis free survival (86.2% and 74.1%, respectively) did not vary with the RT schedule due to the sample size and the uneven distribution of patients. Finally, in intermediate and high risk levels, the type of radiotherapy was not significant and so it did not impact on OS and CSS.

Adjuvant pelvic RT may be associated with acute toxicity, particularly gastrointestinal, genitourinary and haematological side effects and/or late side effects such as vaginal stenosis. In the RTOG0418 [8] and the RTCMIENDOMETRE [9] trials the incidence of gastrointestinal toxicity was around 30%, which depended on the type of treatment and RT techniques. In the present study acute haematological and skin toxicity rates were low (≈ 10% and≈ 20%, respectively) while gastrointestinal and genitourinary toxicity were more marked (43.6% and 35.1%, respectively), although remaining of low grade. IRT alone was associated with almost zero gastrointestinal toxicity and approximately 20% genitourinary toxicity, in accordance with other reports [8, 9].

Toxicity pattern analysis showed that EBRT followed by boost, mainly SIB, was associated with more acute gastrointestinal toxicity. On the other hand, IRT alone had a much lower impact due to the smaller volume that was irradiated.

The incidence of late toxicity, particularly vaginal stenosis was higher in patients treated with EBRT + sequential boost (59.1%) and lower in patients treated with EBRT + IRT (20.4%) or IRT (16.2%). This result is in accordance with literature varying between 10% for G1-2 RTOG scores to 14% for complete vaginal stenosis [10–12].

The main strength of the present study was its focus on radiation therapy in EC and its impact on outcomes. Previous Italian retrospective studies on EC [13–17] did not focus on radiotherapy and, unlike the present study, recruited only small cohorts. Indeed, our large data-base. With 1848 patients accrued in a 10-year period, partially compensated for the retrospective nature of the present analysis, particularly as it had a low rate of missing data, with more than of 75% of information available for oncological outcomes and toxicity. Furthermore, all eligible patients that were enrolled in the database before FIGO 2018 staging system came into routine use, were re-staged according to it so as to achieve homogeneity in tumour classification.

Lack of molecular data may be considered a major limitation of the present study. Since they were, however, proposed only in 2020 obtaining molecular data was not possible within the study time-frame (2010–2020).

However, the growing availability of immunohistochemistry markers analysis can represents an excellent starting point for extending the study retrospectively enrolling the patients with available molecular data and then to built prospective trial.

In this way molecular markers should be considered according to clinical scenario [18, 19] and we will go towards a personalized and more targeting medical therapy.

## Conclusions

This retrospective study provided a picture of Italian use of adjuvant radiotherapy for endometrial cancer in light of current guidelines. External beam and vaginal interventional radiation therapy remain integral aspects of adjuvant therapy for endometrial cancer but molecular study are necessary to personalize the treatment and avoid over- or under-treatment.
